# Detection of Parkinson's Disease Through Automated Pupil Tracking of the Post-illumination Pupillary Response

**DOI:** 10.3389/fmed.2021.645293

**Published:** 2021-03-25

**Authors:** Thasina Tabashum, Adnaan Zaffer, Raman Yousefzai, Kalea Colletta, Mary Beth Jost, Youngsook Park, Jasvinder Chawla, Bruce Gaynes, Mark V. Albert, Ting Xiao

**Affiliations:** ^1^Department of Computer Science and Engineering, University of North Texas, Denton, TX, United States; ^2^Edward Hines Jr. VA Medical Center, Hines, IL, United States; ^3^Department of Ophthalmology, Loyola University Chicago Stritch School of Medicine, Maywood, IL, United States; ^4^Department of Biomedical Engineering, University of North Texas, Denton, TX, United States

**Keywords:** Parkinson's disease, pupil tracking, PIPR, Kalman filter, biomarker

## Abstract

Parkinson's disease (PD) is one of the most common neurodegenerative disorders, but it is often diagnosed after the majority of dopaminergic cells are already damaged. It is critical to develop biomarkers to identify the disease as early as possible for early intervention. PD patients appear to have an altered pupillary response consistent with an abnormality in photoreceptive retinal ganglion cells. Tracking the pupil size manually is a tedious process and offline automated systems can be prone to errors that may require intervention; for this reason in this work we describe a system for pupil size estimation with a user interface to allow rapid adjustment of parameters and extraction of pupil parameters of interest for the present study. We implemented a user-friendly system designed for clinicians to automate the process of tracking the pupil diameter to measure the post-illumination pupillary response (PIPR), permit manual corrections when needed, and continue automation after correction. Tracking was automated using a Kalman filter estimating the pupil center and diameter over time. The resulting system was tested on a PD classification task in which PD subjects are known to have similar responses for two wavelengths of light. The pupillary response is measured in the contralateral eye to two different light stimuli (470 and 610 nm) for 19 PD and 10 control subjects. The measured Net PIPR indicating different responsiveness to the wavelengths was 0.13 mm for PD subjects and 0.61 mm for control subjects, demonstrating a highly significant difference (*p* < 0.001). Net PIPR has the potential to be a biomarker for PD, suggesting further study to determine clinical validity.

## Introduction

Parkinson's disease (PD) is a progressive neurodegenerative disorder. The primary symptoms are muscle weakness, tremor, and rigidity and these are the results of decreased stimulation of the motor cortex arising from the basal ganglia (1). Secondary symptoms may include high-level cognitive dysfunction and speech problems (2, 3). It is crucial to diagnose PD patients as the sufferers start showing physical signs after 70–80% of dopaminergic cells are lost (4). Finding PD early in patients is especially crucial as undiagnosed people experience a neurodegenerative effect, severely lowering the quality of life and increasing mortality (5).

There is no single, well-established biomarker for a definitive diagnosis, suggesting the use of a series of more specific biomarkers (6). Visual dysfunction may result from altered levels of retinal dopamine associated with PD leading to abnormalities such as loss of contrast sensitivity and color perception (7–9). Non-motor complications of PD include various sleep problems such as insomnia, excessive daytime sleepiness, and abnormal sleep-wake cycles that result in poor quality of life (10, 11). Although PD patients who complain of sleep disturbance often qualify for a diagnosis of insomnia, they occasionally demonstrate other primary sleep disturbances such as REM Sleep Behavior Disorder (RBD) or Periodic Limb Movements of Sleep (PLMS) and Restless Legs Syndrome (RLS) (12). Many factors contribute to PD related sleep disturbance however the diurnal nature of PD related sleep disorder implicates circadian dysregulation as a mediator of PD related sleep disturbance. The role of circadian disruption as a correlate and potential etiology component of PD has recently been strengthened by the finding that a key ocular correlate of circadian entrainment has proven causal links to PD related symptomatology (13). The role of circadian dysfunction as an etiologic component of PD has been reinforced by recent studies of Willis et al. (14), who showed that PD related symptomatology can be modified by direct pharmacologic therapy to the retina. These intriguing studies have implicated the retina not only as an associated organ system affected by PD but as a component of the disease etiology (14, 15).

Circadian disorder in PD has been observed by reduced circulating melatonin in PD patients compared with controls (16). It has been proposed that decreased amplitude of melatonin rhythm in PD may result from dysfunction of the suprachiasmatic nucleus (SCN) and/or its afferent and efferent pathways (16). Historically, all phototransduction within the mammalian retina was considered to originate in classical photoreceptors, i.e., rods and cones. However, recent studies have shown that a certain subset of retinal ganglion cells that innervate the suprachiasmatic nuclei (SCN) are in fact themselves photosensitive, and provide an essential function in the entrainment of the circadian clock (17–19). The action of intrinsically photosensitive retinal ganglion cells (ipRGCs) among mammals, including humans, appears based on an opsin termed melanopsin that contrasts to rhodopsin and cone opsins found in conventional photoreceptors (18). Melanopsin expression in the rat retina has been found to be orchestrated by dopamine, possibly *via* dopamine D2 receptors located on ipRGCs (20). Data indicate that dopamine controls melanopsin expression, indicating that classical photoreceptors may modulate the transcription of ipRGC melanopsin production (20). Sakamoto et al. showed that dopamine upregulates melanopsin mRNA expression. Thus, the loss of dopaminergic amacrine cells in PD is expected to cause a reduction in melanopsin expression, and consequently the PIPR. Abnormality of dopamine release by photoreceptors due to PD is expected to impact ipRGC function however the nature of the relationship between loss of retinal dopamine noted and detriment of ipRGC function is unknown. Five morphological subtypes of ipRGCs have been demonstrated in mice (21), and (22) discovered a sixth type of ipRGC. The M1 subtype is involved in non-image-forming visual functions and drives behaviors as the pupillary light reflex and circadian photoentrainment, other types appear to contribute to image-forming as well as non-image-forming visions (23). The relevance of the other subtypes of ipRGC in humans is largely speculative.

The action of intrinsically photosensitive retinal ganglion cells (ipRGCs) among mammals, including humans, appears based on an opsin termed melanopsin that contrasts to rhodopsin and cone opsins found in conventional photoreceptors (18). A key feature of melanopsin driven ipRGC is demonstration of a persistent pupillary response following extinguishing a 480 nm stimulus. This persistent pupillary response is the basis of clinical non-invasive assessment of ipRGC function. In the absence of ocular comorbidity such as glaucoma, the reduction of a persistent pupillary response connotes abnormality of the ipRGC function. Previous studies have shown quite elegantly that abnormalities of ipRGC are highly correlated with defective pupil response at stimuli of precisely 480 nm while stimuli of longer wavelengths (610 nm) resulting from stimulation of photoreceptors are preserved (24, 25). Remarkably, pupils of eyes of humans who were totally “blind” due to X-linked retinitis pigmentosa, a disorder that results in isolated loss of photoreceptors, still responded to stimuli of 480 nm as result of retained activity of the now identified ipRGCs while pupil response to 610 nm (arising from classical photoreceptors) was completely absent (25). In essence, one can separate out ipRGC function by isolating their peak sensitivity (i.e., ~480 nm) and compare findings to retinal sensitivity of alternative wavelengths such as 610 nm. Moreover, in order to determine early-stage PD, Joyce et al. (26) gave evidence that melanopsin and the rod/cone-photoreceptor contribute to pupil control pathways and PIPR can be used as a measurement for the initial assessment of PD. Thus, through spectral sensitivity measurements, we can easily ascertain ipRGC function by appropriate choice of stimulation wavelengths required to induce pupillary constriction.

The relationship between PD and irregular functioning of the retina provides an opportunity to develop a cost-effective and user-friendly system for measuring a PD biomarker. Measuring the rate and extent of change in pupil size and maximal pupil constriction at each stimulus wavelength is tedious for medical professionals. This is an exceedingly time-consuming process that requires evaluation of each single video frame and is thus prone to error. Assessment of ipRGC action now requires expensive equipment coupled with advanced interpretative mechanisms. The intent of our work is to demonstrate the utility of a point of care (POC) ipRGC analytic system easily and effectively deployed in any eye clinic or eye examination facility with little expense. The cost for our system: Ximena firewire camera: $700, LED infrared stimulator: $15, Narrow band path filter: $200, Radiometer: $700, Halogen stimulator: $200. We used our existing slit lamp biomicroscope (used every day for eye examinations) and simply attached the camera to it by the C mount. That is what makes this such a straightforward system, all eye clinics have a slit lamp biomicroscope, we simply adapted ours for about $2k for the testing in conjunction. We have implemented an automated system for tracking pupil diameters over time, making adjustments as needed, and simplifying extraction of parameters such as PIPR can substantially change the clinical accessibility of such a biomarker.

Kalman filters provide a robust and accessible means of tracking parameterized objects in video by combining uncertain frame-by-frame estimates with uncertain state estimates in a Bayesian framework (27). This allows clinicians to directly observe the quality of the fit frame by frame and return parameters of interest for clinical evaluation if desired. Although the work is preliminary, the UI development and integrated tracking are unique and valuable. Kalman filters have been used for pupil tracking. Zhu et al. (28) developed two-staged algorithms to track the eyes by combining the Kalman filter and mean shift. Chi et al. (29) proposed a novel pupil tracking method and utilized a kalman filter to estimate pupil parameters to improve accuracy. The tracking of pupil direction and size has a variety of applications, and Kalman filters provide a robust means of estimating size over time in a variety of contexts. Commercial eye tracking systems are currently available (30, 31), however, our proposed system is tailored toward this application and is available as lightweight, inexpensive software.

In this study, we have developed a real-time prototype that can simultaneously extract pupil size over time, enable adjustment frame by frame, and enable the extraction of experimental parameters to detect Parkinson's Disease. We have collected data for 19 PD and 10 control subjects to assess the abnormality of PD ipRGC function through the post-illumination pupil response (PIPR). The 19 PD subjects represent a range of PD severity with a mean Hoehn and Yahr rating of 2.3 and standard deviation of 1.2. In the H&Y rating system, 1 is for unilateral motor manifestations, 2 is bilateral with minimal effect to balance, 3 and 4 are progressively more severe and affecting balance, while 5 is unable to walk unassisted (32). The produced software implements a pupil tracker with an integrated Kalman filter to measure the abnormal function of ipRGCs in patients with the potential to assist clinicians in the identification of PD.

## Materials and Methods

### System Design

The program automates the process of tracking pupil contraction and dilation by extracting data frame-by-frame and fitting an ellipse on each frame image to track the pupil's size. The system was built using the Python programming language and the graphical user interface was written in PyQt5. To begin the process, after uploading the video, the user is prompted to click on a region inside the pupil of the image to get a center point of the pupil, and the rest of the process is automated. Radial lines from the selected center point are drawn in 30° arcs. The program computes the intensity gradient along each ray. Maximal gradients are points on the edge of the pupil. The length is averaged out to get the diameter of the pupil and then the program fits an ellipse around the pupil for each frame. This center and ellipse are then used as an initial estimator for the subsequent Kalman filter sequence.

**Figure 1:**
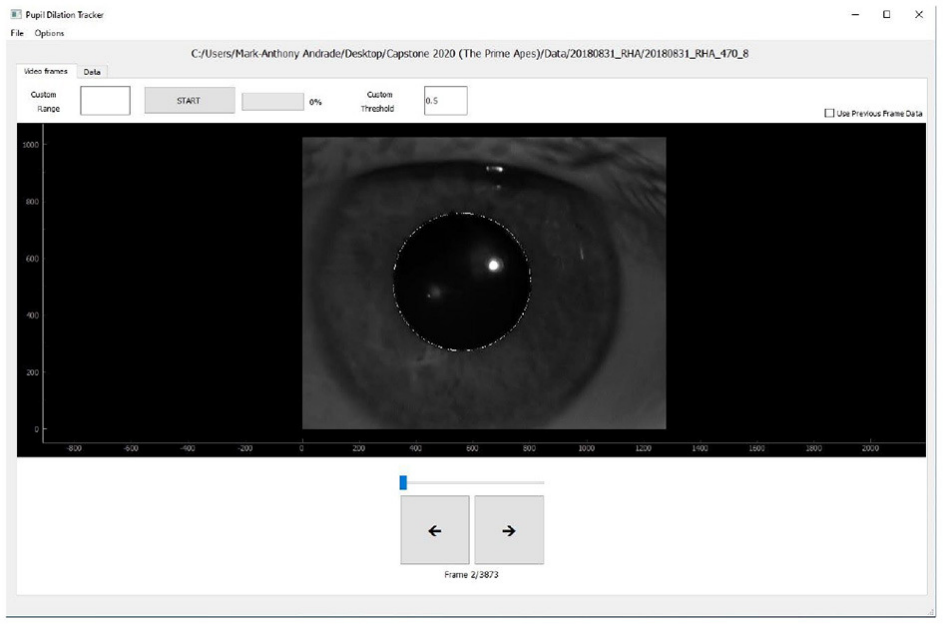
User interface for the developed pupil tracking system.

After fitting one frame, successive frames can be fit by a button press, with an optional choice of time span to automate before stopping. The program will save pupil size and location parameters for viewing and are also available for easy download for offline analysis. Figure 1 demonstrates the user interface after uploading the video file. The user can scroll through all the frames and can observe the pupil size and location estimate of a frame by clicking on it. The user can further adjust the estimates and even tune the thresholds the Kalman filter relies on to make the automated process more reliable depending on their data. This software is available upon request to the corresponding author, and will be released as open source software upon further development.

### Kalman Filter Estimation

The Kalman filter is an efficient method to estimate the state of a system over time under uncertainty. In this framework, the state of the system is best represented as a Markov process with conditional independence from prior history when the parameters are known. Because the radius of the pupil shifts relatively slowly over the duration of the experiment, we model the pupil through only the radius (R) and the center position (X_c_ and Y_c_) as both variables change slowly. There are two steps in the Kalman filter process - the prediction step and the update step, governed by Bayesian update rules as a linear quadratic estimator. We have set the initial center position based on the user input, and the initial radius of the pupil is estimated as described using the maximum change in the luminance gradient for a series of radial lines. The system tracks the covariance of the estimates in position and size over time along with the observation noise of R = 1 the process noise **Q = 0.0002** which were fit by observation over a set of trials. In this study, we have adopted Fixed-Lag smoothing with *N* = 4 (33), which allows us to produce smoothed estimates for fixed smoothing windows.

### Data Collection

Patients were enrolled for the study by consecutive sampling. Inclusion criteria included a prior diagnosis of PD with treatment of one or more dopaminergic agonists and an age of 65 or older. Patients taking drugs known to impact circadian rhythm including lithium, benzodiazepines, steroidal, and non-steroidal anti-inflammatory drugs and vitamin B12 were excluded from study. Patients were screened prior to testing for presence of ocular abnormalities including, amblyopia, cataract reducing correctable vision to <20/25 in either eye, glaucoma, or any form retinopathy such as related to diabetes or age-related macular degeneration. Degree of PD severity was evaluated by the Hoehn & Yahr rating system (34).

Subjects were secured in a completely dark room for 10 min prior to ipRGC assessment to facilitate dark adaptation. Pupillometry assessment was conducted in dim red room illumination to preserve the dark-adapted state during pupillometry testing. Each study subject was allowed to dark adapt for a 10 min period prior to ipRGC testing. Dark adaptation occurred following eye dilation (so at the end of the 20 min dilation period) and in the same room in which ipRGC analysis was performed. The right eye was dilated with 2.5% phenylephrine and 1% tropicamide. Dilation of the right eye was employed in an effort to maintain consistent retinal illumination within and between subjects during stimulation. After a 20-min dilation period, the ipRGC driven pupil response was measured *via* the consensual post-illumination pupil response (PIPR) in the left eye. Stimuli presented to the right eye consisted of long-wavelength (red) and short-wavelength (blue) narrowband 5-s pulses of light. This establishes the adequacy of the irradiance level used in this study to induce ipRGC action. Light stimuli were generated using a halogen-based fiber optic light source coupled with a custom-built Maxwellian-view optical system consisting of narrow bandpass filters of 25 mm diameter short wavelength, “blue” light, λmax = 470 nm; full width half maximum (FWHM) = 10 nm and long wavelength, “red” light, λmax = 640 FWHM = 10 nm) imaged to the plane of the pupil in conjunction with appropriate neutral density filters to alter power output to 8 or 30 μW (ILT 9000, International Light Technology). The stimulus pencil was constrained using an aperture of 10 mm in diameter. Each subject was run through three trials for each stimulus combination of 470 and 610 nm at both 30 μW high energy and 8 μW low energy. The average of three trials are used for analysis and the trials in which the blinking eyes are removed. While the stimulus is introduced into the dilated right pupil, the left pupil is concurrently videotaped. Each trial was run for 40 s: 5 s of pre-stimulus baseline, the light stimuli was then presented to the right pupil for 5 s, and recording continued for 30 s post-stimulus. Data was collected from a total of 24 PD and 11 control subjects. Among them 22 patients with Parkinson's disease have clinically confirmed PD, while two subjects were demonstrating parkinsonism symptoms - they are included as PD in this study. Two subjects were demonstrating parkinsonism symptoms due to presumed Lewy body dementia and multiple system atrophy, respectively.

The experimental setup included use of an infrared sensitive XIMEA MQ013RG-E2 machine vision camera whose parameters were controlled by the bundled XIMEA CamTool software, which also enables initiation of photo and video capture. Patients were placed securely in the biomicroscope to maintain alignment in Maxwellian view while the dilated right eye was stimulated. The stimuli for both long and short wavelengths were determined based on a spectral irradiance of 8 and 30 μW. cm^−2^ nm^−1^ resulting in irradiance stimuli of 11.421 and 11.995 log photons cm^−2^ s^−1^ at 640 and 14.621 and 15.195 log photons. cm^−2^ s^−1^ at 470 nm determined at the corneal plane, respectively. Given the older age of the participants, retinal irradiances were estimated based upon established corrections for age-related changes in the optical density of the media of the eye for stimuli >3° in diameter (35). The pupillary light reflex was determined by averaging three consensual pupil recordings of 40 s, (5 s pre-stimulus, 5 s stimulus and 30 s post-stimulus). Pupillary dynamics were assessed using the Ximea CamTool software in conjunction with Adobe Premier Pro v.2 video capture and ImageJ imaging software (Rasband, W.S., ImageJ, U. S. National Institutes of Health, Bethesda, Maryland, USA, http://rsb.info.nih.gov/ij/, 1997-2007). Nerve fiber layer thinning has been associated with PD, therefore assessment of retinal nerve fiber layer thickness and retinal morphology was assessed in the dilated stimulated eye using automated optical coherence tomography (Zeiss Cirrus Model 5000 OCT, Carl Zeiss AG, Oberkochen, Germany) (36).

**Figure 2:**
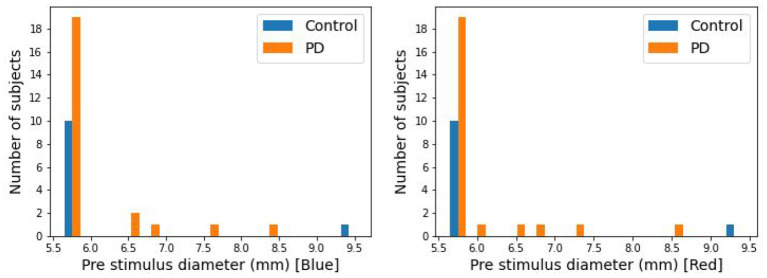
Distribution of pre-stimulus pupil diameter.

The pupillary responses of 24 PD and 11 control subjects were recorded in this study. It has been observed that PIPR substantially correlates with baseline pupil diameter (37), leading to a potential confound in this limited study. 29 of the 35 subjects had pre-stimulus pupil diameters between 5.5 and 5.7 mm (Figure 2). Five subjects had pupil diameters above 5.7 mm (5 PD and 1 control). In this present study for the analysis purpose those 29 subjects have been used.

### Data Analysis

The frame rate is 30 fps. The pupil diameters from all subject videos were extracted and stored for analysis. The stored files are then analyzed using python. We defined the pre-stimulus diameter as the average pupil diameter, over a 5-s period, before the light stimulus. Post-stimulus diameter was the average pupil diameter for the 30 s after the light stimulus. The measured parameters used to observe a difference between PD and control are post-illumination pupil response (PIPR) and Net PIPR Net PIPR.

PIPR = Pre-stimulus Pupil Diameter – Post-stimulus Pupil Diameter.

Net PIPR = Blue PIPR – Red PIPR.

We also elaborated on these measures by normalizing by the pupil diameter creating the following additional measures: PIPR% and Net PIPR%.

PIPR Percentage = (PIPR^*^100)/Pre-stimulus Pupil Diameter.

Net PIPR Percentage = [Blue PIPR Percentage – Red PIPR Percentage].

The mean and standard error for each population are calculated for all the parameters, and the PIPR and Net PIPR of the two groups are compared by the Student *t*-test. Additionally, Pearson correlation was used to compare Net PIPR to levodopa equivalent dosage, age, and PD severity.

## Results

**Figure 3:**
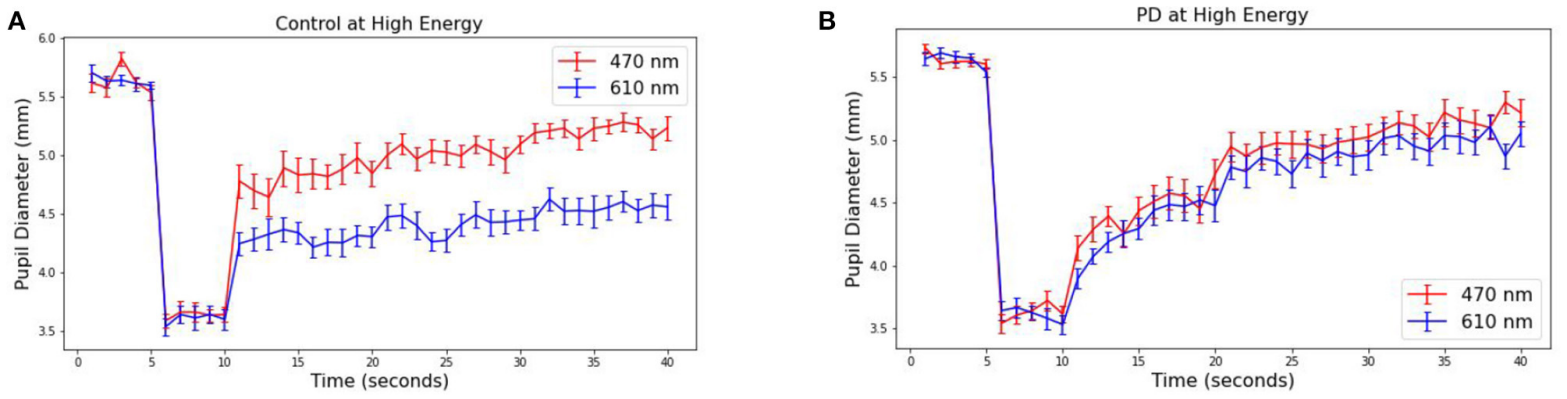
Average pupillary response (±SEM) to blue (470) and red (610) light for **(A)** Control and **(B)** PD subjects at 30 μW.

**Figure 4:**
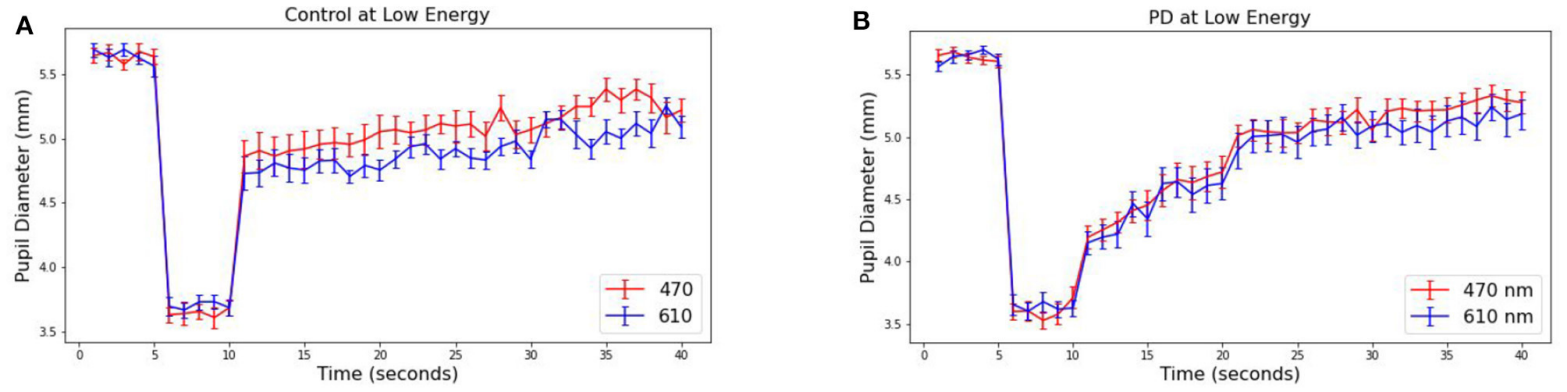
Average pupillary response (±SEM) to blue (470) and red (610) light for **(A)** Control and **(B)** PD subjects at 8 μW.

The pupillary response is known to differ between red and blue wavelengths in non-PD subjects but is similar among patients with Parkinson's disease (26, 38). To observe this in our collected data, the average pupil diameter of 19 PD and 10 control subjects are plotted over time (Figure 3 at 30 μW and Figure 4 at 8 μW). Table 1 includes 19 patients with Parkinson's disease age, levodopa equivalent dosage, and Hoehn & Yahr rating system at the time of study enrollment. Subject 115 and 116 are patients with parkinsonism symptoms.

**Table 1:** Description of 19 patients with Parkinson's disease.

**Subject #** **mean (±std)**	**Age (years)** ** 70.84 (±5.9)**	**LEDD (mg)** **925.68 (±864.8)**	**PD Grade** ** 2.31 (±1.2)**
101	65	750	2
102	69	300	3
103	65	240	1
104	68	600	3
105	85	1,197	3
106	67	450	1
107	72	1,390	3
108	66	580	1
109	74	300	1
110	66	2,760	4
111	66	1,995	3
112	80	1,995	2
113	81	640	3
114	64	600	3
115	73	0	1
116	72	450	1
117	73	214	4
118	70	2,827	4
119	70	300	1

The pre-stimulus diameters for patients with Parkinson's disease were 5.63 mm for PD and Control subject groups, different by no more than 0.01 mm in either group or stimulus condition prior to light exposure. For the 10 control subjects, the average blue (470 nm) post-stimulus diameter was 4.42 (±0.08) mm, and the average red (610 nm) post-stimulus diameter was 5.02 (±0.08) resulting in a difference of 0.60 mm (*p* < 0.00001). For the 19 patients with Parkinson's disease, the average blue post-stimulus diameter is 4.72 (±0.08) mm and the average red post-stimulus diameter is 4.84 (±0.08), resulting in a difference of only 0.12 (*p* = 0.3). Notably, there also appears to be a visible difference in the time course of the average response after stimulus offset in PD compared to control subjects for both wavelengths of light.

**Figure 5:**
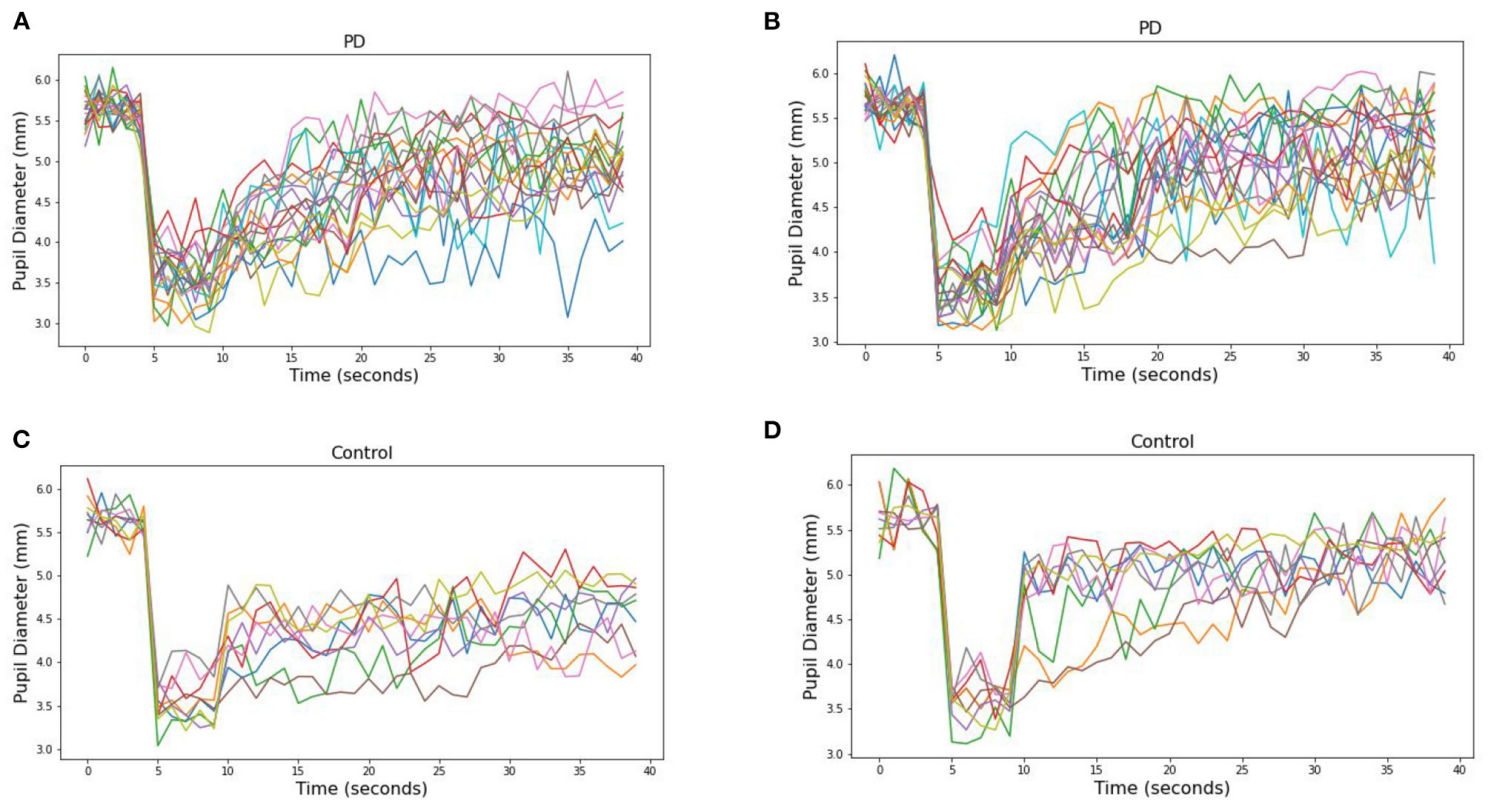
Pupillary response to **(A)** blue (470) and **(B)** red (610) light for each PD subjects at 30 μW and **(C)** blue (470) and **(D)** red (610) light for each Control subjects.

From these measured pupillary responses, the descriptive measurement of PIPR, Net PIPR, and Net PIPR Percentage were performed for high and low energy. In Table 2, the mean and std values for the parameters at 30 μW measures are shown. The student *t*-test analysis of Net PIPR and Net PIPR Percentage for control vs. PD demonstrated a highly significant difference between groups (*p* < 0.001). This is in contrast to average PIPR parameters for each wavelength which only showed no significance (*p* < 0.14 red) or minimal significance (*p* < 0.03 blue) that is expected as it is not using subject contrast. A similar analysis was performed for the low energy (8 μW) stimulus showing that Net PIPR, as well as PIPR (blue/red), are not found to be statistically significant at low energy (*p* > 0.05). Figure 5 illustrates time trace plots for each participant at high energy to understand the variability between the subjects.

**Table 2:** Pupillary response parameters and significance testing to discriminate between PD and Control subjects.

**Parameters**	**PD**	**Control**	***P*-value**
PIPR (blue) (mm)	0.92 (±0.08)	1.22 (±0.08)	*P* = 0.03
PIPR (red) (mm)	0.79 (±0.08)	0.61 (±0.07)	*P* = 0.14
Net PIPR (mm)	0.13 (±0.08)	0.61 (±0.05)	P^*^ < 0.001
Net PIPR percentage (%)	2.35 (±1.35)	10.82 (±0.93)	P^*^ < 0.001

* *Significant p values*.

**Figure 6:**
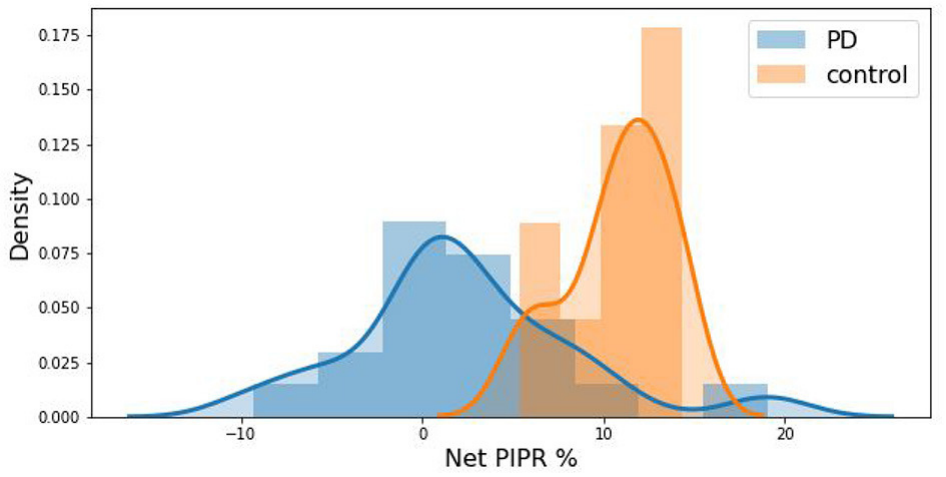
Kernel density estimation curve depicting the distribution of subjects by Net PIPR Percentage indicating a difference between PD (blue) vs. Control (red).

**Figure 7:**
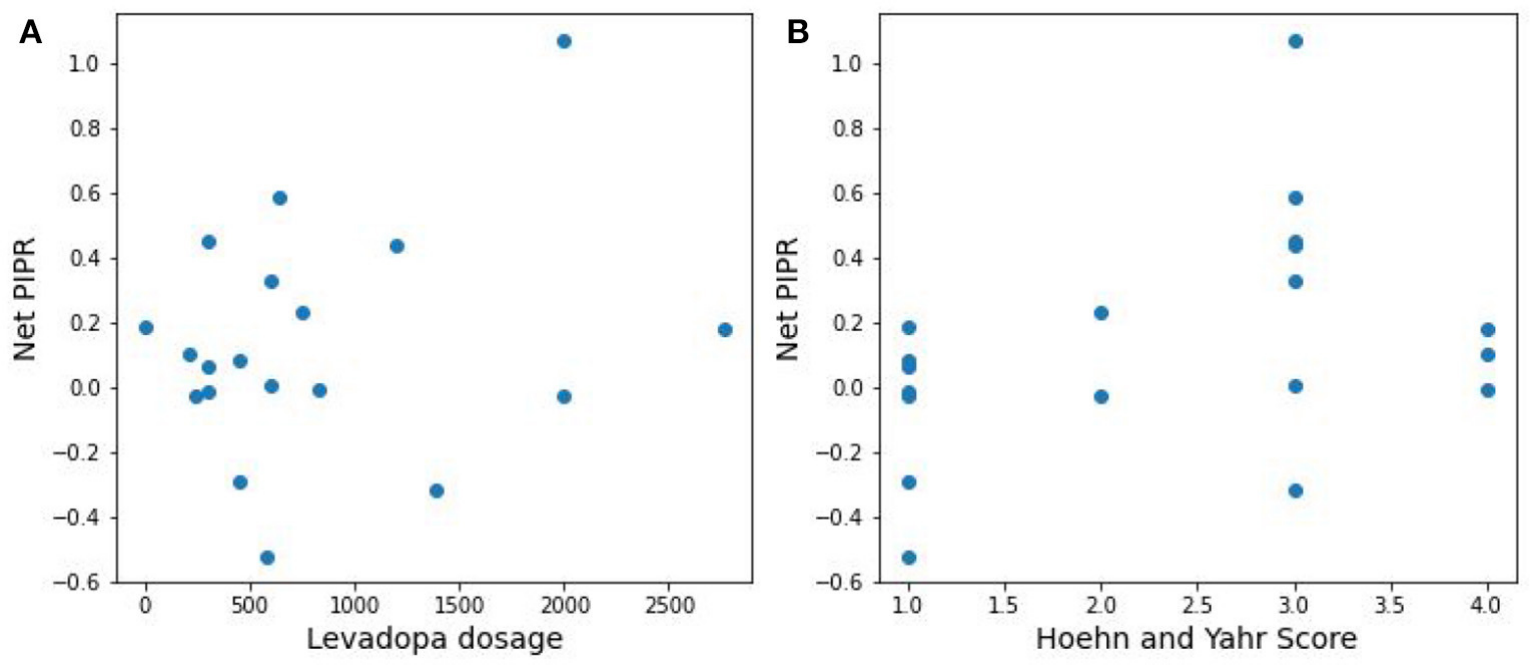
Scatter plots of the relationship between Net PIPR and **(A)** levodopa dosage and **(B)** PD severity.

The distribution difference between PD vs. Control is illustrated in Figure 6 by using a probability density curve. In addition to distinguishing PD from control subjects, the nature of the relationship between PD severity and net PIPR was explored. PD severity was measured through the Hoehn and Yahr (H&Y) score as well as the subject's current Levodopa Equivalent Dosage (LED). There was no clear statistical significance relationship found given this sample size and variability. The correlation coefficient for Net PIPR with H&Y score was 0.384 (*p* = 0.1) while the correlation between Net PIPR and LED was 0.237 (*p* = 0.3). The relationships can be observed among all patients with Parkinson's disease analyzed in Figure 7.

## Discussion

Our results show a significant difference in Net PIPR values between patients with Parkinson's disease and controls - The average Net PIPR in PD (0.13 mm) is significantly smaller than control subjects (0.61 mm). This supports earlier research indicating PD patients have a reduction in the sustained pupil responses, and suggests this parameter can be used as a biomarker for Parkinson's disease. Because all subjects chosen for analysis had approximately the same baseline pupil diameter, there was not a substantial difference in discriminability between Net PIPR and Net PIPR Percentage, reporting similar discriminability between PD (2.35%) and controls (10.82%) for Net PIPR Percentage. Notably, the two individuals with suspected PD demonstrated reduced Net PIPR consistent with patients with Parkinson's disease with values of −0.3 and 0.2, providing further evidence of their initial diagnosis.

The analyses in this paper focused on the subjects with a similar baseline pupil diameter as has been observed in other studies (37). A similar analysis was performed for all 35 subjects, including the 5 PD and 1 control subject with baseline pupil diameters greater than the 29 subjects between 5.5 and 5.7 mm. Notably, statistical significance of Net PIPR and Net PIPR% are both less profound when all baseline diameter sizes of the subject are included (*p* < 0.03 all sizes vs. *p* < 0.001 under 5.7 mm). Kankipati et al. (37) investigated the relationship between Net PIPR and baseline pupil diameter and demonstrated Net PIPR positively correlated with baseline pupil diameter. Joyce et al. (39) showed that pupillary response is affected by baseline diameter when expressed in mm, but it can be decorrelated when normalized. However, it would be interesting to collect sufficiently varied data with a range of baseline pupil diameters for further study. Also, the use of Net PIPR Percentage only minimally counteracts the effect of this expanded range of baseline pupil diameters (*p* < 0.02 all sizes vs. *p* < 0.001 under 5.7 mm) suggesting that adequately controlling for pre-stimuli diameter of the subjects may be a crucial factor to using this biomarker.

Previous studies indicated that sustained pupil constriction primarily occurs as a result of the response of intrinsically photoreceptive retinal ganglion cells (24). Joyce et al. demonstrated the reduction of PIPR in PD that is associated with impaired pupil pathways with changes in pupil diameter was measured in percent relative to baseline. For an equivalent comparison, we normalized baseline pupil diameter and found that the difference in Net PIPR (*p* < 0.01) was minimally significant compared to Net PIPR in mm (*p* < 0.001). Joyce et al. measured PIPR 6 s after the light offset as previous studies (40–42) showed that rhodopsin and melanopsin contribute to the ipRGCs responses at early redilation stage which is primarily attributed to melanopsin in our study. On the basis of that interpreting our result solely as melanopsin is challenging. However, pupil responses can differ by underlying condition, light wavelength, and experimental setup.

In this study, we examined the discriminability of Net PIPR and its potential as a biomarker for disorders affecting photoreceptors, such as Parkinson's Disease. Net PIPR and Net PIPR Percentage for distinguishing control vs. PD demonstrated show a high level of statistically significance (*p* < 0.001) establishing the proposed parameters can be a metric to determine PD. The results of this study will inform a larger trial examining ipRGC function in various forms of parkinsonism related disorders and support the long term goal of identification of suitable retinal biomarkers for neurologic diseases implementing light-based PD therapy. However, further studies are needed to evaluate the hypothesis as our investigation is performed with small sample size and a limited range of pupil sizes.

## Conclusion

In this study, control subjects display a distinct but minimally overlapping Net PIPR compared to PD indicating its potential utility as a biomarker for PD consistent with the known melanopsin mechanism. The analyses were performed using a custom-built pupil-size tracking system for acquired video under repeated exposure to red and blue wavelengths of light in the contralateral eye. The robust user-interface facilitated direct observation of pupil-size fits, manual adjustments as needed, and recomputing of future fits to enable clinicians to efficiently extract the relevant parameters for Net PIPR. The analyses demonstrate the potential utility of this experimental paradigm and our online pupil-size tracking system improves clinical efficiency and accessibility as a diagnostic tool in evaluating patients with suspected Parkinson's disease.

## Data Availability Statement

The raw data supporting the conclusions of this article will be made available by the authors, without undue reservation.

## Ethics Statement

The studies involving human participants were reviewed and approved by IRB, Edward Hines, Jr. VA Hospital. The patients/participants provided their written informed consent to participate in this study.

## Author Contributions

TT, MA, and TX created the pupil tracking application and analyzed the data. AZ, RY, KC, MJ, YP, JC, and BG designed the experimental protocol and performed subject data collection. TT, BG, MA, and TX wrote the paper. All authors contributed to the article and approved the submitted version.

## Conflict of Interest

The authors declare that the research was conducted in the absence of any commercial or financial relationships that could be construed as a potential conflict of interest.
